# Active break program: an evidence- based review for the promotion of occupational health

**DOI:** 10.47626/1679-4435-2025-1488

**Published:** 2025-11-17

**Authors:** Wagner Vieira Sant’Ana, Alexandre Simas de Medeiros

**Affiliations:** 1 Universidade Castelo Branco, Physical Education, Rio de Janeiro, RJ, Brazil.; 2 Universidade Federal do Rio de Janeiro, Transportation Engineering Program, Rio de Janeiro, RJ, Brazil.

**Keywords:** occupational health, ergonomics, health policy, labor gymnastics, saúde ocupacional, ergonomia, política de saúde, ginástica laboral

## Abstract

The objective of this study is to scientifically organize research on the impacts of short active breaks on employees’ physical and mental health, with the aim of proposing a model adapted to the Brazilian work environment. The narrative analysis is structured around five main themes: (1) types of exercises recommended in the workplace context; (2) evidence of cholesterol reduction and physiological benefits; (3) differentiation by age group and biotype; (4) effects on stress and mental health; and (5) additional benefits associated with outdoor physical activity. Clinical trials and longitudinal studies published between 2010 and 2024 were analyzed. Active breaks proved effective in lowering LDL, improving HDL, and controlling stress (*variabilidade da frequência cardíaca *and cortisol), relieving musculoskeletal pain, and increasing physical and emotional vitality. Activities performed outdoors intensified the positive psychophysiological effects. The findings highlight the feasibility of implementing short breaks, guided by specialists and tailored to employee profiles, as an ergonomic and preventive strategy, in accordance with Regulatory Standard No. 17 and the Occupational Health Medical Control Program. The regular adoption of active breaks is a low-cost, high- impact measure for promoting health in the workplace.

## Abstract (pt)

O objetivo desta pesquisa é organizar cientificamente estudos sobre os impactos das pausas ativas de curta duração na saúde física e mental dos empregados, com o propósito de sugerir um modelo que se adapte ao cenário laboral do Brasil. A análise narrativa está organizada em cinco temas principais: (1) tipos de exercícios recomendados no contexto laboral; (2) evidências de redução de colesterol e benefícios fisiológicos; (3) diferenciação por faixa etária e biotipo; (4) efeitos sobre o estresse e saúde mental; e (5) benefícios adicionais associados à prática de atividades ao ar livre. Analisaram-se ensaios clínicos e pesquisas longitudinais divulgadas entre 2010 e 2024. As pausas ativas se mostraram efetivas na diminuição da lipoproteína de baixa densidade, no aprimoramento da lipoproteína de alta densidade e controle do estresse (variabilidade da frequência cardíaca e cortisol), no alívio de dores musculoesqueléticas e no aumento da disposição física e emocional. As atividades realizadas ao ar livre intensificaram os impactos positivos na psicofisiologia. Os resultados destacam a possibilidade de implementar pausas curtas, orientadas por especialistas e ajustadas ao perfil dos empregados, como estratégia ergonômica e preventiva, conforme estabelecido na Norma Regulamentadora nº 17 e no Programa de Controle Médico de Saúde Ocupacional. A adoção de pausas ativas frequentes é uma ação de baixo custo e grande impacto para a promoção da saúde no ambiente de trabalho.

## INTRODUCTION

The increasing demand for productivity in the workplace has increased physical and emotional stress among workers, making it essential to implement strategies that promote health, well-being, and quality of life during working hours. Among these strategies, active breaks – particularly those organized in short intervals, such as 15-minute sessions – have proven effective in reducing stress levels, improving cardiovascular health, and promoting psychophysiological balance.

The notion of occupational health, understood as the state of physical, psychological, and social well-being of workers while performing their professional duties, goes beyond the mere elimination of work-related diseases to also include aspects of prevention, health promotion, and ergonomics. Studies indicate that brief interventions, combined with regular physical activity in the workplace, may contribute to reducing stress indicators such as cortisol and heart rate variability (HRV). In addition, they can help control cholesterol levels, preventing chronic diseases, and increase job satisfaction.^1,2^

Short active breaks are defined as planned intervals during the workday in which employees interrupt prolonged periods of sedentary behavior to perform light-intensity exercises – such as stretching or short walks – with the aim of reducing sedentary behavior and increasing physical activity, while also potentially promoting health benefits and improving work engagement.^3^ While active breaks are primarily advocated for their positive effects on the health and well-being of employees, evidence also indicates that wellness interventions can generate measurable financial returns for organizations. Structured workplace wellness programs show an average return on investment ratio of 3:1, primarily due to reduced medical expenses and lower absenteeism rates. Moreover, evidence suggests that for every dollar invested in workplace wellness, organizations can save up to $2.73 in absenteeism costs and $3.27 in health-related expenses. Therefore, investments in supervised exercise sessions may result in substantial gains in productivity, reduced absenteeism, and a strengthened organizational environment.^4^

Despite the growing interest in active break programs, there remains a lack of systematization of evidence linking different exercise modalities to measurable workplace outcomes, such as pain, stress, cardiovascular health, and productivity. Moreover, analyses that include contextual variables, such as body type and natural environment, are still scarce when evaluating the effectiveness of these interventions. Consequently, the existing body of literature offers only a limited practical framework for the development of personalized, evidence-based health programs.

This study is relevant to the field of occupational safety and health engineering because the proposal to implement organized active breaks in the workplace is directly aligned with Regulatory Standard No. 17 (NR-17),^5^ established by Ordinance No. 3.214/1978 of the Brazilian Ministry of Labor and Social Security. Item 17.6.4 of NR-17 establishes that work activities involving muscular overload or uncomfortable/awkward postures must include breaks or other measures to mitigate adverse health effects. Thus, the implementation of brief active breaks during work represents both a technical and organizational prevention strategy, in full compliance with regulatory guidelines and supported by current scientific evidence.

The proposal discussed in this article is also aligned with the principles of the Occupational Health Medical Control Program (*Programa de Controle Médico de Saúde Ocupacional*, PCMSO), as established by NR-7,^6^ providing support for health promotion initiatives that help prevent work-related diseases, such as musculoskeletal disorders, chronic stress, and psychosocial hazards. The inclusion of short and regular exercise sessions during the workday can be incorporated into the action plans developed by occupational health teams as an educational and collective measure, particularly in contexts that demand high cognitive effort or involve repetitive strain. Thus, this review not only fills a scientific gap but also contributes to the development of preventive strategies in the workplace, promoting increased well-being and safer, healthier environments that comply with Brazilian labor regulations. Thus, the objective of this study was to systematically synthesize the existing scientific evidence on interventions involving short exercise sessions performed during the workday, with the goal of determining which activities – typically lasting around 15 minutes – are most effective in promoting workers’ health. The study was structured around five main topics: (1) exercise modalities recommended in workplace settings; (2) evidence of cholesterol reduction and other physiological benefits; (3) variations according to age group and body type; (4) impacts on stress and mental health; and (5) additional benefits associated with performing outdoor and nature-based activities.

## METHODS

The aim of this narrative literature review was to identify and synthesize scientific evidence on the effects of different types of physical activity in the workplace, with the objective of proposing an active break program focused on promoting the physical and mental health of workers. The methodology employed combines thematic analysis of the studies with the practical application of the findings, considering criteria such as feasibility, safety, and workplace adaptation. This narrative review was reported according to the Preferred Reporting Items for Systematic Reviews and Meta-Analyses (PRISMA), with adaptations made to suit the thematic and qualitative scope of the study.

### SEARCH STRATEGY AND SELECTION CRITERIA

Study selection was conducted between March and June 2025 through manual and targeted searches in the PubMed, ScienceDirect, SpringerLink, and Google Scholar databases, with additional citation tracking (snowballing). Combinations of descriptors in Portuguese and English were used (e.g., “*atividade física no trabalho*,” “*ginástica laboral*,” “active breaks,” “microbreaks,” “work stress,” “cortisol,” “HRV,” “nature- based interventions”).

Inclusion criteria were: (i) empirical studies (clinical trials, longitudinal, or quasi-experimental designs) and systematic reviews/meta-analyses with detailed methodological descriptions; (ii) published between 2000 and June 2025; (iii) in Portuguese, English, or Spanish; (iv) involving adults aged 18–65 years in occupational settings or with direct workplace applicability; and (v) exercise-based interventions reporting measurable physiological, psychological, or functional outcomes.

Purely theoretical or opinion articles without empirical data, studies focused on pediatric or high- performance sports populations, research unrelated to occupational contexts, studies with insufficient methodological descriptions, and those without access to the full text were excluded.

Classic instrument validation studies were cited only to substantiate the measurement tools and were not included among eligible studies.^7,8^

### THEMATIC ORGANIZATION OF THE STUDIES

The selected articles were grouped into themes representing the main dimensions of the relationship between physical activity and occupational health. The thematic groups guided both the analytical process and the subsequent practical proposal: Theme 1 – Active breaks and microbreaks (pain, fatigue, and well-being); Theme 2 – Physical training programs and lipid profile; Theme 3 – Prevention and control of work-related musculoskeletal pain; Theme 4 – Stress and mental health (cortisol, HRV, well-being); Theme 5 – Outdoor exercise and contact with nature.

Each thematic group included five distinct articles, ensuring methodological diversity, variability of context, and thematic robustness.

### ANALYSIS PROCEDURES

All studies were read in full and analyzed according to the following criteria: (i) study objective and target population; (ii) type, intensity, and duration of the intervention; (iii) setting of application (indoor, outdoor, workplace, or clinical); (iv) measured variables (eg, cortisol, HRV, pain, cholesterol, quality of life); (v) main results and conclusions; and (vi) applicability and replicability in other work environments.

For each article, an interpretive paragraph was written summarizing the study’s central contribution, methodology, main findings, and concluding remarks on its potential application in the design of an active break program.

### PRACTICAL APPLICATION OF THE FINDINGS

The findings from the analysis of the five thematic groups served as the basis for the development of a structured model for a workplace physical activity program. This model integrates multiple goals – stress reduction, cardiovascular improvement, pain prevention, personal adaptation, and interaction with nature – into a plan tailored to specific occupational settings. The proposed framework is presented and discussed in the next section, including recommendations on frequency, duration, target population, and implementation strategies.

## RESULTS

### LITERATURE REVIEW: FOUNDATIONS AND THEMES

Understanding the effects of brief exercises in the workplace requires a multifactorial approach, given the diversity of worker profiles, work environments, and physiological responses involved. Although the scientific literature on active breaks in the workplace has expanded over recent decades, it remains fragmented across distinct thematic areas, which hinders the formulation of integrated, evidence-based recommendations. In this context, the present literature review was organized into five interdependent thematic groups, each representing a critical dimension of the relationship between occupational health and brief physical activity.

The first thematic group addresses the types of exercise recommended in the workplace, considering factors such as intensity, duration, complexity, and biomechanical safety. The second group focuses on evidence related to the reduction of cholesterol levels and other physiological markers (eg, blood pressure and blood glucose) associated with the practice of brief exercise. The third group examines variations according to age group, body type, and physical condition, discussing how interventions should be adapted to different profiles. The fourth group explores the effects of physical activity on stress and mental health indicators, with emphasis on hormones such as cortisol and measures of psychological well-being. Finally, the fifth group investigates the contribution of outdoor exercise and nature-based interventions (NBIs), which have been increasingly associated with enhanced emotional and physiological benefits.

The systematic synthetization of these five thematic groups is essential for advancing the development of effective, safe, and scientifically grounded protocols for active breaks in the workplace. Each thematic group provides a specific lens through which to examine the complex interface between movement, health, and the work environment, allowing managers, occupational health professionals, and researchers to make decisions more closely aligned with both empirical evidence and the needs of different groups of workers. Therefore, this literature review aimed to map the most relevant scientific contributions within each of these areas, identifying existing gaps, points of convergence, and directions for future research.

### BRIEF EXERCISE THAT HELPS REDUCE CHOLESTEROL LEVELS AND IMPROVE CARDIOVASCULAR HEALTH

Labecka et al.^7^ conducted a randomized controlled trial (RCT) evaluating the effectiveness of an active break intervention on nonspecific low back pain in young adults, with two groups (n = 25 per group). The intervention consisted of performing lumbar and hip extension exercises every 30 minutes over a 12-week period. A significant reduction was observed in pain (visual analogue scale [VAS]), low back discomfort (Borg scale), and functional disability (Oswestry Disability Index) in the experimental group, with the effects maintained over time. The study validated a simple, reproducible, and applicable protocol for school and work environments involving young populations, representing a nonpharmacological alternative for managing low back pain associated with prolonged sitting posture.^7^

Akkarakittichoke et al.^8^ conducted a three-arm cluster-RCT to investigate the effects of active break and postural shift interventions on the recovery duration and recurrence of neck and low back pain in 193 office workers. Participants used a smart device for 12 months that prompted breaks or postural shifts throughout the workday. The intervention groups showed a significant reduction in recovery time and a lower recurrence rate compared with the control group, with adjusted hazard ratios of 0.22 for active breaks and 0.35 for postural shifts. The study highlights the potential of continuously applied workplace technologies as effective strategies for the recovery and prevention of recurrent musculoskeletal pain.^8^

Radwan et al.^9^ conducted a systematic review analyzing several studies on the effects of active microbreaks on sedentary workers. The results demonstrated consistent benefits in reducing musculoskeletal discomfort, fatigue, and mental stress, without compromising productivity. Effective protocols involved short and frequent breaks (typically every 30 minutes) with light-intensity activities such as walking, stretching, or joint mobility exercises. The study consolidated the concept of the “active microbreak” as an accessible and effective ergonomic tool for promoting physical and mental health across different occupational settings.^9^

Albulescu et al.^10^ performed a meta-analysis of 22 independent study samples (n = 2,335) to investigate the effects of microbreaks on well-being (vigor and fatigue) and work performance. The results showed small but statistically significant effects of microbreaks in boosting vigor (d = 0.36; p < 0.001) and reducing fatigue (d = 0.35; p < 0.001). Although no significant effects were found on overall performance (d = 0.16; p = 0.116), positive impacts were observed for tasks requiring lower cognitive demand. These findings reinforce the role of brief breaks of up to 10 minutes as a strategy for the momentary recovery of affective and cognitive resources, particularly in activities requiring sustained attention, consistent with the Conservation of Resources Theory and the Effort-Recovery Model.^10^

### PHYSICAL TRAINING PROGRAMS AND LIPID PROFILE (AEROBIC, STRENGTH, OR COMBINED)

Smart et al.^11^ conducted an extensive systematic review with meta-analysis of 148 RCTs involving 8,673 participants, with the aim of determining the impact of different types of exercise on major lipid markers: total cholesterol, high-density lipoprotein cholesterol (HDL-C), low-density lipoprotein cholesterol (LDL-C), triglycerides, and very low-density lipoprotein cholesterol (VLDL-V). Statistical analyses employed meta-regression and Trial Sequential Analysis, allowing control for type I and type II errors and assessment of sample size adequacy. The results showed that exercise training – particularly combined training (aerobic + resistance) – significantly reduced LDL-C levels (-7.22 mg/dL) and triglycerides (-8.01 mg/dL), while increasing HDL-C levels (+2.11 mg/dL). The main contribution of the study was to demonstrate, with high statistical robustness, that regular exercise can serve as an alternative or complement to pharmacological interventions in the management of dyslipidemia, providing substantial benefits for cardiovascular health.^11^ Stanton et al.^12^ conducted a RCT investigating the effects of different exercise intensities on lipid profile and cholesterol effiux capacity in overweight adults. Over a 12-week period, 33 participants completed three weekly sessions of either moderate- or high-intensity exercise, with detailed analysis of lipoprotein composition and HDL functionality. Both groups showed significant increases in HDL-C and cholesterol effiux capacity, particularly in the HDL2 subfractions, with greater effects observed in the high-intensity group. The study demonstrated that even relatively short bouts of exercise, when performed at adequate intensity, are sufficient to produce meaningful metabolic benefits with clinical relevance for the prevention of atherosclerosis.^12^

Elsayyad et al.^13^ examined the effects of 8 weeks of moderate aerobic exercise on the lipid profile of 50 healthy female university students. The protocol involved three weekly treadmill sessions with controlled duration and intensity. At the end of the intervention, significant reductions were observed in total cholesterol, LDL-C, VLDL-C, and triglyceride levels, along with a substantial increase in HDL-C. The study reinforces the effectiveness of short and accessible physical activity programs for young adults, even in populations without evident risk factors, contributing to early preventive strategies against dyslipidemia and cardiovascular disease.^13^

Kraus et al.^14^ conducted one of the most influential studies on the relationship between the intensity and amount of exercise and lipid profile. The research involved 111 overweight participants assigned to groups that performed different amounts and intensities of walking or running over a 6-month period. The results showed that even moderate-intensity and lower amounts of exercise (eg, walking 12 miles per week) produced significant reductions in LDL-C and improvements in the total cholesterol/HDL-C ratio. The study demonstrated that prescribing accessible and sustainable workouts is sufficient to improve lipid profiles, supporting their adoption as a public health strategy for cardiovascular disease prevention.^14^

### PREVENTION AND CONTROL OF WORK- RELATED MUSCULOSKELETAL PAIN

Andersen et al.^15^ conducted a RCT to evaluate the effectiveness of specific resistance training for reducing neck and shoulder pain among workers exposed to high levels of repetitive tasks. The implementation of interventions involving isometric exercises resulted in a statistically significant reduction in pain, particularly among participants who demonstrated higher adherence to the protocol. The study showed that targeted exercises with progressive load, even when performed in lower amounts, have a therapeutic effect on occupational muscle pain.^15^

Izquierdo et al.^16^ developed international guidelines for prescribing exercise in older adults, emphasizing multicomponent interventions that integrate strength, balance, and flexibility. The recommendations are based on a comprehensive review of RCTs and demonstrate that individualized interventions using appropriate volume and intensity are essential for preventing functional decline and musculoskeletal pain. The article provides strong evidence supporting therapeutic exercise as an effective nonpharmacological approach for older adults.^16^

Noormohammadpour et al.^17^ investigated, in a single- blind RCT involving nurses with chronic low back pain, the effects of an 8-week multistep core stability exercise program consisting of weekly supervised sessions and home exercises. Pain (VAS), disability (Roland–Morris), quality of life (Short Form-36), and the diameter of lateral abdominal muscles (via ultrasound) were evaluated. The intervention group showed a significant reduction in pain (≈30 points on the VAS), improved functional capacity and quality of life (except for the emotional role domain), and increased muscle thickness during contraction. These results indicate that a supervised, progressive program is effective in mitigating musculoskeletal pain among nursing professionals.^17^

Suorsa et al.^18^ investigated the association between physical fitness (estimated VO?peak on bicycle ergometer, modified push-up test, sit-up test), physical function (chair-rise test, maximal walking speed), and work ability (Work Ability Score) among aging public- sector workers. Positive associations were found between VO?peak, push-up performance, and walking speed with work ability, while an inverse relationship was observed between chair-rise time and work ability. These findings suggest that maintaining physical fitness can support work capacity in this age group (cross-sectional study; no pain assessment included).^18^

In a cluster-RCT, Johnston et al.^19^ evaluated the impacts of multimodal exercise interventions in the workplace among administrative employees. In the secondary outcome analysis, reductions in neck pain and improvements in functional capacity were observed, both associated with adherence to the program. The results support the feasibility of supervised active breaks as an effective strategy to alleviate musculoskeletal pain in corporate environments.^19^

### STRESS AND MENTAL HEALTH (CORTISOL, HEART RATE VARIABILITY, WELL-BEING)

Cheema et al.^20^ investigated the effects of a worksite- based yoga program on HRV in physically inactive adults. Over a 10-week period, participants engaged in regular *hatha *yoga sessions during their lunch breaks. The results indicated a significant increase in HRV and a reduction in perceived stress markers, suggesting that integrative, low-impact exercise programs are effective in improving workers’ mental and physiological health, even with time restrictions and in occupational settings.^20^

Brinkmann et al.^21^ compared the effectiveness of HRV biofeedback and mindfulness meditation among administrative employees experiencing occupational stress symptoms. Using an experimental design with three groups, the study found that the biofeedback group exhibited greater increases in HRV and greater reductions in perceived anxiety than the mindfulness and control groups. These findings suggest that physiologically oriented interventions, such as biofeedback, may provide superior measurable benefits in workplace contexts, contributing to evidence-based strategies for stress management.^21^

Wicks et al.^22^ showed that engaging in physical activity in natural environments, compared with urban environments, is associated with greater psychological benefits, including reduced anxiety and fatigue and increased positive affect and vigor, although some heterogeneity was observed across studies. In light of well-established theoretical models on attention restoration and stress recovery through contact with nature, the authors concluded that incorporating natural elements into active break programs may enhance their mental health benefits.^22^

Tonello et al.^23^ conducted a systematic review examining the relationship between physical activity, HRV, and work-related stress. Based on seven longitudinal studies, they found that workers with higher physical fitness levels exhibited greater HRV, indicating a higher capacity for stress management. The study’s main contribution lies in proposing HRV as a useful biomarker for monitoring the effectiveness of physical interventions targeting mental health, underscoring the potential of structured programs to promote physiological resilience.^23^

Van Dijk et al.^24^ conducted a randomized exploratory pilot study with Swedish workers to examine the effects of mechanical massage and mental training on biological indicators of physiological stress. Although no significant differences in HRV were observed in the overall sample, a reduction in plasma cortisol was detected among participants with elevated systolic blood pressure. Stratified analysis revealed that responses to the interventions varied according to baseline physiological conditions, reinforcing the importance of individualized programs for promoting mental health in the workplace.^24^

### OUTDOOR EXERCISE AND CONTACT WITH NATURE

Antonelli et al.^25^ conducted a systematic review and meta-analysis to evaluate the effects of “forest bathing” (walking in a forest environment) on cortisol as a stress biomarker. Twenty-two studies were included, eight of which were part of the meta-analysis. The results showed significantly lower salivary cortisol levels among participants exposed to forest environments compared with urban environments, both before (mean difference [MD] = −0.08 μg/dL; 95%CI −0.11 to −0.05) and after the intervention (MD = −0.05 μg/dL; 95% CI −0.06 to −0.04), indicating short-term stress reduction. The authors noted that anticipatory or placebo effects may have contributed to the results and recommended further research.^25^

Gritzka et al.^26^ conducted a systematic review focusing on workplace NBIs and found evidence – from RCTs and other longitudinal pretest–posttest designs – that planned contact with green spaces in occupational settings is associated with improvements in mental health and well-being, including reductions in perceived stress and fatigue, with reports of favorable psychophysiological effects (HRV and cortisol) in some studies. The authors suggest that corporate policies and practices incorporate regular exposure to nature as a complementary strategy for workplace health promotion.^26^

Song et al.^27^ compared the effects of a single walking session conducted in natural versus urban environments, monitoring both psychological and physiological parameters. The results showed that walking in a forest led to greater emotional recovery and lower heart rate. The methodology included pre- and post-intervention measures in healthy participants. The main practical implication is that short breaks in natural environments can be strategically incorporated into the workday to promote relaxation and well-being, even without the need for long-term structured interventions.^27^

Jones et al.^28^ mapped experimental and quasi- experimental studies on greenspace interventions, such as walking in parks, gardening, and horticultural therapy, focusing primarily on stress markers (cortisol). A general trend toward reduced physiological stress was observed in association with these interventions across different populations, including workers, although methodological heterogeneity limited definitive conclusions. The authors recommend incorporating NBIs as a complement to existing psychosocial strategies within occupational settings.^28^

Calogiuri et al.^1^ conducted a pilot study in Norway involving workers who participated in a green- exercise intervention – light-intensity physical activity performed outdoors in natural environments. Based on psychometric assessments performed before and after the intervention, the researchers observed a significant reduction in perceived stress among participants. The main contribution of the study lies in demonstrating that even short exposures to natural environments during active breaks at work can reduce occupational stress, representing a practical and cost-effective alternative for companies to promote mental health in the workplace.^1^

### SUMMARY OF MAIN FINDINGS

Given the extensive and high-quality body of scientific evidence presented in this review, the key results that stand out and warrant emphasis are summarized below.

Multifactorial effectiveness of active breaks: several studies demonstrated that microbreaks and active breaks have consistent positive effects on musculoskeletal pain, fatigue, and work-related vigor (as shown in RCTs and systematic reviews). Improvements in lipid profiles (↓LDL, ↓triglycerides, ↑HDL) resulted from regular physical activity programs (aerobic, strength, or combined) conducted over periods ranging from weeks to months, along with enhanced cholesterol effiux capacity and reduced musculoskeletal pain. Clinical trials^12,15^ validate the potential of these interventions as effective nonpharmacological measures.

Positive impact on mental health and stress: some studies^23,25^ revealed that regular physical activity, particularly when performed outdoors, significantly reduces cortisol levels, increases HRV, and enhances psychological well-being. The literature demonstrates that these practices produce both immediate and long- term benefits, especially when tailored to workers’ individual profiles and specific occupational contexts.

Importance of adaptation to functional profiles: evidence from works such as the study by Izquierdo et al.^16^ and Suorsa et al.^18^ underscores that individualized interventions, considering factors such as age, physical condition, and type of work, tend to be more effective. Multicomponent exercises that combine strength, flexibility, and balance are recommended for older workers, while resistance and mobility exercises provide targeted benefits for populations experiencing work- related pain.

Additional benefits of contact with nature: the fifth thematic group is particularly innovative in demonstrating that physical activity performed in natural environments amplifies both emotional and physiological benefits, as shown by some studies.^27,28^ Outdoor walks, breaks in green spaces, and activities under sunlight are associated with greater relaxation, reduced stress reactivity, and improved emotional recovery.

### PROPOSAL FOR AN ACTIVE BREAK PROGRAM IN THE WORKPLACE

Based on the evidence systematized in this literature review, a weekly active break program is proposed below, consisting of approximately 15-minute sessions that integrate warm-up exercises, adapted calisthenics, and cool-down techniques focused on breathing and stretching. The proposal adheres to the ergonomic guidelines of NR-17^5^ and the principles of the PCMSO,^6^ making it applicable across a variety of work environments, including those with limited space or infrastructure.

The selected physical activities are based on evidence from studies demonstrating the benefits of brief exercise sessions for improving musculoskeletal health, managing stress, regulating physiological parameters, and fostering overall well-being. The program is structured to ensure biomechanical safety, ease of implementation, and suitability for individuals of different ages and fitness levels, as highlighted throughout the thematic analysis.

The weekly subdivision presented in Table 1 introduces variety to prevent monotony, encourage employee engagement, and promote diverse physical stimuli throughout the workday. The suggested exercises may be modified by occupational health specialists based on prior physical fitness assessments and individual limitations. Table 1 provides a detailed outline of the daily sessions, including estimated duration, notes on accessibility, and recommended environmental conditions.

**Table 1:** Weekly workplace exercise program (15–20 minutes per day)

Day of the week	Stage	Proposed exercises	Duration (min)	Notes
Monday	Warm-up	Marching in place + shoulder and hip rotations	3	Adaptable for seated position
	Calisthenics	Squats (with or without support), wall push-ups, wall plank	10	10–12 repetitions each
	Cool-down	Cervical and thoracic stretches + 4–6 s breathing	5	May include relaxing music
Tuesday	Warm-up	Light walking in the hallway (or in place) + arm circles	3	Alternative: light jumping jacks
	Calisthenics	Alternating knee raise, horizontal arm extension with band, wall bridge	10	Suitable for all age groups
	Cool-down	Standing lower limb stretches + diaphragmatic breathing	5	Natural ventilation recommended
Wednesday	Warm-up	Cross crawl marching + ankle rotations	3	Can be performed seated
	Calisthenics	Sit-to-stand from chair, trunk rotation, light desk plank	10	Well accepted by older adults
	Cool-down	Scapular stretches + guided breath counting	5	Reduces cervical tension
Thursday	Warm-up	Light brisk walking + shoulder lifts	3	Focus on posture
	Calisthenics	Wall push-ups, supported squats, alternating side steps	10	Can be performed in groups
	Cool-down	Quadriceps and lower-back stretches + mindful breathing	5	Effective for chronic pain relief
Friday	Warm-up	Stick mobility (broom handle) + dynamic stretching	3	Add light background music
	Calisthenics	Partial squats, resistance band row, lateral arm raises	10	More playful and varied
	Cool-down	General stretching + silent breathing-focused pause	5	End-of-week relaxation

### PROPOSED MONITORING AND EVALUATION OF THE WORKPLACE ACTIVE BREAK PROGRAM

To evaluate the effectiveness of the proposed workplace active break program, it is recommended that functional, physiological, and psychosocial assessments be performed before the start of the activities (baseline) and after 12 weeks of consistent practice (reassessment). The objective is to identify potential improvements in functional capacity, physical parameters, and postural stability through practical, low-cost procedures. These assessments can be conducted directly in the workplace by qualified professionals (Table 2).

**Table 2:** Functional physical evaluation

Test	What it assesses	How to apply	Reference
30-Second Chair Stand Test	Lower-limb strength and endurance	Number of repetitions completed in 30 seconds	Jones et al.^29^ - functional assessment for older adults
Modified push-up (1 min)	Upper-limb strength	Number of push-ups performed against a wall or on knees	Hassan^30^
Upper-limb flexibility	Flexibility and joint mobility	Touch fingers behind the back	Back Scratch Test^31^
Single-leg balance (eyes open and closed)	Postural stability	Seconds maintained standing on one leg	Balance Test^32^
2-Minute Walk Test	Cardiorespiratory endurance	Total distance covered	Butland et al.^33^

To improve functional assessment, it is recommended to use validated tools that allow for the measurement of subjective perceptions of pain and physical well-being in the workplace. These instruments can be administered independently or with the assistance of an occupational safety technician, nurse, or occupational health professional, providing valuable information for the continuous monitoring of workers’ health (Table 3).

**Table 3:** Subjective pain assessment

Instrument	What it measures	How to apply	Reference
Pain Visual Analogue Scale	Intensity of pain in the back, shoulders, and knees	Rating from 0 to 10	Pain Scale^34^
Work Ability Index	Perceived health and work ability	Validated questionnaire	Martinez et al.^35^

The psychophysiological dimension of workplace well-being, as shown in Table 4, can be assessed using standardized tools that capture both the subjective components of perceived stress and the objective indicators of autonomic state. Ideally, these instruments should be administered by a psychologist or another qualified professional to ensure data validity and the confidentiality of responses, particularly within corporate environments.

**Table 4:** Psychological assessment of well-being

Instrument	What it measures	Details	References
Perceived Stress Scale	Everyday stress	10 questions, 5-point Likert scale	Cohen et al.^36^
WHO-5 Well-Being Index	Positive mood indicators	5 items, scale 0–25	Topp et al.^37^
Heart rate variability	Autonomic balance/physiological stress	Measured with a simple heart rate monitor (RMSSD)	Task Force^38^

RMSSD = root-mean square differences of successive R-R intervals; WHO = World Health Organization.

The evaluation of the effectiveness of a workplace active break program should also include occupational variables, as shown in Table 5, which allow for the quantification of direct effects on employees’ work routines and perceptions. These indicators complement physical, physiological, and psychological assessments, providing management with both objective and subjective information about the organizational impacts of the intervention.

**Table 5:** Attendance

Indicator	How to measure	Frequency
Absenteeism	Number of days missed due to health reasons	Compare pre- and post-intervention
Presenteeism	Self-report or specific questionnaire	After 12 weeks
Satisfaction with the program	0–10 rating scale (anonymous feedback)	Monthly

The comparison of data collected at baseline (T0) and after 12 weeks of intervention (T1) allows direct evaluation of the program’s impact on employees. For this purpose, statistical analyses of means and medians from physical assessments, pain scales, and stress measures are recommended. The paired Student’s *t*-test (for normally distributed variables) and Wilcoxon test (for nonparametric data) are appropriate to identify significant variations between the two time points. The interpretation of results should consider not only statistical significance but also clinical and occupational indicators of functional improvement, such as increased endurance, reduced reported pain, and enhanced psychophysiological balance.

Beyond point-in-time assessments, continuous monitoring of the program is essential to ensure its long-term effectiveness. A quarterly review of the data is recommended to allow for adjustments in the prescribed activities based on individual responses – whether for progressive load increase, maintenance, or modification due to physical limitations. This dynamic process also allows the reorganization of groups according to age, functional status, or specific goals, thereby maximizing adherence and program benefits. In summary, the proposed strategy integrates scientific evidence, operational feasibility, and ergonomic adaptation, establishing a sustainable model for workplace health promotion.

The implementation of the PDCA cycle (Plan – Do – Check – Act) emerges as an effective strategy to ensure quality, continuous monitoring, and adaptability of the workplace active break program. During the planning phase, the main goal is to promote improvements in employees’ physical and mental health through frequent, evidence-based active breaks. This phase involves creating a weekly task schedule, selecting assessment methods (physical, physiological, and psychological), and organizing employee groups according to their functional abilities. The implementation phase consists of the daily execution of the planned exercises under professional supervision, with ongoing adjustments to workplace conditions as needed.

In the verification phase, results obtained before and after 3 months of practice (T0 and T1) are compared using physical assessments, pain and stress scales, HRV, and performance indicators such as absenteeism and job satisfaction. Based on this evaluation, the final phase of the cycle (ie, Act) allows for modifications in the type and intensity of activities, group organization, and implementation logistics. This iterative methodology ensures both the effectiveness and feasibility of the program, allowing its institutionalization as a permanent component of workplace health promotion and ergonomic policies.

## DISCUSSION

This study proposed a structured active break program and a multifaceted evaluation method, both of which are supported and complemented by recent scientific evidence, particularly the studies by Santos et al.^39^ and Batista-Ferreira et al.^40^. While the former provides a broad evidence base through a systematic review, the latter validates the proposal through a proof- of-concept study conducted in a real-world occupational setting, confirming that the approach presented here is theoretically sound, practically feasible, and effective for promoting occupational health.

The efficacy of active breaks, one of the central themes of this study, is strongly corroborated by the findings of Batista-Ferreira et al.^40^ In a study involving administrative employees, a 25-week intervention led to a substantial reduction in sedentary behavior (from 31% to 14% of participants sitting more than 10 hours per day), a decrease in physical inactivity (from 43% to 26%), and significant declines in perceived stress and bodily pain (p < 0.01). The high adherence rate (64%) further reinforces the feasibility of integrating short, frequent workouts into the workday to achieve measurable health benefits.

From a broader perspective, the systematic review by Santos et al.^39^ analyzed 15 high-quality studies and concluded that workplace physical activity interventions are effective in reducing musculoskeletal pain and the use of analgesics. However, the review also emphasized that no single strategy proves universally effective, highlighting the heterogeneity of existing protocols and the need for multidimensional approaches, such as participatory ergonomics, to ensure both worker engagement and the success of the interventions.^39^

The multifactorial and personalized approach advocated in the present study is also supported by the cited studies. The demonstrated need for active management programs^40^ and interventions that integrate physical and psychosocial components^39^ represents a key point of convergence. This alignment reinforces the proposal to tailor interventions to functional profiles, combining exercise routines with strategies that promote mental well-being and organizational engagement.

Finally, the proposed evaluation system – which integrates functional tests, pain scales, psychological assessments, and the PDCA cycle for continuous monitoring – is consistent with best practices in occupational health research. It reflects both the pre- and post-intervention evaluation methodology employed by Batista-Ferreira et al.^40^ and the use of validated instruments (such as the Nordic Musculoskeletal Questionnaire and the VAS), as well as the emphasis on long-term follow-up highlighted in the review by Santos et al.^39^

Comparison with recent literature confirms that the implementation of active breaks, even in remote work settings, is a viable and effective strategy. The physiological and regulatory foundations of this approach – with documented benefits for HRV, cholesterol regulation, and compliance with NR-17 and PCMSO guidelines –consolidate the proposed program as a robust, evidence-based intervention for promoting health and well-being in the workplace.

This study has some limitations. The studies included in the literature review had significant methodological heterogeneity, such as differences in intervention duration and intensity, which may affect the comparability of results. Moreover, many samples were not representative of diverse body types, age groups, or occupational settings, highlighting the need for caution when interpreting these findings.

The main contribution of this study lies in the systematic and comparative integration of evidence that allows the formulation of practical guidelines for short active breaks, tailored to employees’ profiles and the available physical environment. This approach promotes a healthier workplace that is better equipped to meet everyday demands.

## CONCLUSIONS

The organization of themes into five groups allowed the identification of consistent and convergent evidence regarding the benefits of brief active breaks for employees’ physical and mental health. The clinical studies, reviews, and meta-analyses examined indicate that even short-duration interventions, when implemented in a structured and consistent manner, produce tangible effects in reducing musculoskeletal pain, improving cardiovascular indicators, and mitigating occupational stress. This body of evidence provides the technical foundation for recommending an intervention program adaptable to a variety of work environments.

The distinct contribution of this research lies in its combination of a detailed literature review with the proposal of a scientifically grounded and operationally adaptable 15-minute active break protocol. This approach fills a significant gap in the literature, which often presents isolated findings without translating them into practical models for daily routines in private companies or public institutions. By integrating the rigor of scientific review with a practical methodology for monitoring and evaluation, this study helps bridge the gap between research and professional practice.

The inclusion of basic instruments for physical, psychological, and occupational assessment makes the program accessible to multidisciplinary teams, reducing the need for complex infrastructure and allowing its replication across multiple organizational settings. The use of validated scales, such as the VAS, Work Ability Index, and Perceived Stress Scale, alongside biomarkers such as HRV, improves the program’s capacity to monitor intervention outcomes without compromising operational feasibility. Furthermore, the application of the PDCA cycle for regular follow-up and adjustment establishes the program as a continuous process of organizational learning.

The proposal also stands out for recognizing the importance of adapting exercises according to age, physical condition, and professional role, thereby ensuring safe and effective inclusion of a wide range of employee groups. At the same time, it underscores the role of the environment, particularly contact with nature, as a key factor that amplifies the benefits of physical activity, an aspect increasingly relevant to workplace mental health policies.

The implementation of structured, evidence- based active break programs in the workplace has proven to be an efficient and cost-effective strategy for promoting the physical and mental health of employees, reducing musculoskeletal complaints, and improving overall quality of life. These interventions not only improve workers’ well-being but also have the potential to decrease absenteeism and presenteeism, thereby increasing punctuality and productivity. For organizations, the benefits include reduced occupational health expenditures and the strengthening of a corporate culture centered on prevention and the appreciation of human capital. The consistent application of these practices, coupled with ongoing monitoring and periodic adjustments, can serve as a strategic differentiator in employee care and effective human resource management.

This study provides promising opportunities for future studies, particularly to empirically validate the proposed protocol through longitudinal designs and to evaluate its effects on health indicators, quality of life, and productivity. The implementation of evidence-based active break programs should be regarded not merely as a preventive measure but as a structural investment in promoting workplace well-being and supporting the human sustainability of organizations.
